# Associations Between Cortical Iron Accumulation and Memory in Patients With Amnestic Mild Cognitive Impairment and in Cognitively Normal Individuals

**DOI:** 10.1002/brb3.70521

**Published:** 2025-05-19

**Authors:** Subin Lee, Suhyeon Lee, Ina Park, Yeonsil Moon, Younghee Yim, Jongho Lee, June Sic Kim, Won‐Jin Moon

**Affiliations:** ^1^ Laboratory for Imaging Science and Technology, Department of Electrical and Computer Engineering Seoul National University Seoul South Korea; ^2^ Department of Radiology Konkuk University Medical Center, Konkuk University School of Medicine Seoul South Korea; ^3^ Department of Neurology Konkuk University Medical Center, Konkuk University School of Medicine Seoul South Korea; ^4^ Department of Radiology Chung‐Ang University College of Medicine, Chung‐Ang University Hospital Seoul South Korea; ^5^ Clinical Research Institute Konkuk University Medical Center Seoul South Korea; ^6^ Research Institute of Medical Science Konkuk University of Medicine Seoul South Korea

**Keywords:** iron, memory, mild cognitive impairment, quantitative susceptibility mapping

## Abstract

**Background and Purpose:**

Brain iron accumulation is recognized as a cause and therapeutic target in Alzheimer's disease (AD). We investigated the differences in both volume and iron accumulation between cognitively normal (CN) older adults and patients with amnestic mild cognitive impairment (aMCI). Additionally, we assessed which combination of these measures best explains the group differences in visual and verbal memory performance.

**Materials and Methods:**

We retrospectively analyzed data from 48 patients with aMCI and 33 age‐matched CN individuals. Structural differences were investigated using voxel‐based comparisons of T1‐weighted magnetic resonance images. Differences in iron accumulation were investigated using voxel‐based comparisons of quantitative susceptibility mapping (QSM) images. Subsequently, significant clusters from these voxel‐based analyses (amygdala, posterior cingulate cortex, precuneus, lateral occipital cortex, and pericalcarine cortex) were entered into a stepwise regression to predict verbal and visual memory scores, while accounting for age, sex, and education as covariates.

**Results:**

In comparison to CN, patients with aMCI had significantly lower scores in both verbal and visual memory tests (*p* < 0.001). The T1‐weighted voxel‐based morphometry (VBM) results showed significant hippocampal atrophy in the aMCI group relative to CN individuals. The QSM‐VBM results showed increased iron accumulation in the amygdala, posterior cingulate cortex, precuneus, lateral occipital cortex, and pericalcarine cortex (FWE‐corrected *p* < 0.05). Lower hippocampal volume (*B* = 2015.91, SE = 469.61, *p* < 0.001) and higher posterior cingulate cortex susceptibility (*B* = –189.63 SE = 89.11, *p* = 0.037) were significant predictors of verbal memory. For visual memory, higher lateral occipital susceptibility (*B* = –659. 96, SE = 253.03, *p* = 0.011) was significant imaging predictor.

**Conclusions:**

These results suggest that iron accumulates in regions where atrophy has not yet occurred, suggesting that iron may serve as an earlier imaging marker of neurodegeneration compared to volume atrophy. Further studies are needed to investigate the longitudinal relationship between brain volume and iron accumulation during cognitive decline.

## Graphical abstract

The T1‐weighted VBM analysis revealed significant hippocampal atrophy in the aMCI group. QSM‐VBM showed increased iron accumulation in multiple brain regions (FWE‐corrected *p* < 0.05). Lower hippocampal volume and higher posterior cingulate cortex susceptibility predicted verbal memory, while higher lateral occipital susceptibility predicted visual memory. These findings suggest iron accumulation may precede atrophy, serving as a potential early marker of neurodegeneration.

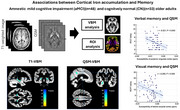

**Abbreviations**
AD: Alzheimer's disease
aMCI: amnestic mild cognitive impairment
*APOE*
: apolipoprotein E gene
CSF: cerebrospinal fluid
MCI: mild cognitive impairment
MRI: magnetic resonance imaging
QSM: quantitative susceptibility mapping

## 1 Introduction

Alzheimer's disease (AD) is a slowly progressing complex age‐related neurodegenerative disorder, and prior to manifesting as clinical dementia, it presents itself in an early stage with only mild cognitive or memory impairments (Jagust 2008). To benefit from early intervention in mildly affected individuals, the identification and definition of a syndrome called mild cognitive impairment (MCI) has been introduced (Flicker et al. 1991; Petersen et al. 1999). Among the heterogeneous types of MCI, amnestic MCI (aMCI) is widely considered a prodromal stage of AD. It is characterized by age‐related objective memory impairment while general cognitive function remains preserved (Petersen 2004). While amyloid/tau/neurodegeneration (ATN) imaging biomarker approaches enable us to identify AD pathology in early stages, determining aMCI in patients remains challenging, especially true when using MRI and limited clinical information, and when advanced amyloid/tau PET and extensive neuropsychiatric tests are not available.

Iron, an essential metal for brain biological functions including neurotransmitter synthesis, myelin generation, and metabolism (Ward et al. 2014), plays an important role in AD. Accumulation of iron can induce oxidative stress, culminating in the death of neuronal cells (Lane et al. 2018). While local brain iron levels increase during normal aging (Hallgren and Sourander 1958; Li et al. 2014), previous studies have suggested that elevated levels are a significant risk factor for AD (Scott Ayton et al. 2015; Duce et al. 2010). A large postmortem cohort study revealed a correlation between brain iron levels and the rate of disease progression in AD (S. Ayton et al. 2021). However, whether iron is a cause or consequence of neurodegeneration remains to be elucidated (Ndayisaba et al. 2019). A recent study suggested that the iron‐dependent programmed cell death pathway (ferroptosis) may play a vital role in neurodegenerative diseases, such as AD (Y. Wu et al. 2023).

Quantitative susceptibility mapping (QSM) is a frequently utilized post‐processing technique that gauges the magnetic susceptibility of tissue specimens through gradient‐echo magnetic resonance imaging (MRI). This technique allows for the estimation of brain iron levels by establishing a relationship between the observed phase and the local magnetic field (Bulk et al. 2018; Langkammer et al. 2012), and provides high sensitivity and specificity for quantifying iron content. While the iron content shows positive magnetic susceptibility, calcium deposition shows negative magnetic susceptibility due to its diamagnetic properties (Yamada et al. 1996). Thus, QSM can be used to study iron accumulation in various neurodegenerative diseases, including AD and MCI (Kan et al. 2022; Kim et al. 2023; Sun et al. 2017; Tu et al. 2022); however, few studies have reported iron concentrations in the prodromal stage of AD, particularly aMCI (H. G. Kim et al. 2017).

In aMCI, the main symptom is episodic memory impairment, specifically involving the verbal and visual memory (Albert et al. 2011). Although episodic memory is mainly processed in the medial temporal lobe, verbal and visual memory involve different neural pathways, with verbal memory involving the left hemisphere, visual memory involving the right hemisphere (Bonner‐Jackson et al. 2015).

We hypothesized that brain iron accumulation extends beyond brain atrophy, affecting a wider brain area, and that specific memory function decline is correlated with iron levels in functionally relevant areas. We assume that cortical iron level is more related to biochemical change in the early stage of neurodegeneration, the result of which is measured by brain atrophy. Therefore, we aimed to evaluate iron accumulation patterns in patients with aMCI and further evaluate their association with memory. We investigated the relationship between regional brain volume and iron accumulation in a cognitively normal (CN) older population and in patients with aMCI. In addition, we evaluated the combination of structural atrophy and QSM values to best explain group differences in verbal and visual memory performance.

## 2 Materials and Methods

This retrospective study (IRB No. 2020‐09‐030) was approved by the Institutional Review Board of our hospital and was performed in accordance with the latest version of the Declaration of Helsinki. The requirement for informed consent was waived because of the retrospective nature of the study.

### 2.1 Participants

We initially considered 109 participants aged 60–80 years from a retrospective registry, who underwent MRI and were diagnosed with aMCI (*n* = 49) or CN (*n* = 60). Among the 109 participants, 81 had available test scores for specific memory functions (visual and verbal). Therefore, 33 age‐matched CN participants and 48 patients with aMCI were included in the final analysis. The 33 CN individuals who underwent 3‐Tesla brain MRI were selected from our previous prospective studyYang J. et al. 2023.

We assessed basic demographic characteristics, apolipoprotein E (APOE) gene mutation status, scores from the Mini‐Mental State Examination (MMSE), values from the Geriatric Depression Scale (GDepS), and results from the Seoul Verbal Learning Test (SVLT) delayed recall (verbal memory) and Rey‐Osterrieth Figure Test (RCFT) delayed recall (visual memory) (Kang et al. 2019). Patients with one or two copies of an APOE e4 allele were defined as APOE e4 carriers. Other pathologies were ruled out using T2 and FLAIR sequences of brain MRI. Diagnoses of MCI were based on the guidelines from Diagnosis and Statistical Manual of Mental Disorders (4th ed.), the criteria of the National Institute of Neurological and Communicative Disorders and Stroke and the Alzheimer's Disease and Related Disorders Association (NINCDS‐ADRDA) (McKhann et al. 1984) along with the criteria set forth by Petersen et al. (1999).

### 2.2 MRI Acquisition

All participants underwent MRI scans using a 3‐Tesla MAGNETOM Skyra unit (Siemens Healthineers, Erlangen, Germany) with a 20‐channel coil. The MR protocol included the following MRI sequences: (1) axial turbo spin echo T2‐weighted imaging, with the following parameters: TR = 4450 ms, TE = 81 ms, flip angle = 150°, matrix size = 512 × 358, FOV = 220 × 220 mm^2^, slice number = 28, slice thickness = 5.0 mm, and gap = 2.0 mm, (2) axial fluid‐attenuated inversion recovery (FLAIR): TR = 9000 ms, TE = 95 ms, flip angle = 150°, matrix size = 320 × 188, FOV = 220 × 220 mm^2^, slice thickness = 5.0 mm with a 2.0 mm gap, (3) coronal 3D T1‐weighted magnetization‐prepared rapid acquisition of gradient‐echo (MPRAGE) sequences: TR = 2300 ms, TE = 2.98 ms, flip angle = 9°, matrix size = 256 × 256, FOV = 256 × 256 mm^2^, slice number = 192.

In addition, multi‐echo axial 3D T2^*^‐weighted gradient‐recalled‐echo (GRE) imaging was performed using the following parameters: TR/TE = 51 ms/8.9 ms, six echoes with echo spacing = 4.09 ms, FA = 20°, bandwidth = 150 kHz, FOV = 240 × 240 mm^2^, matrix = 416 × 312, in‐plane resolution = 0.938 × 0.938 mm^2^, slice number = 60, slice thickness = 2 mm, and acquisition time = 4 min 33 s. This sequence was used to reconstruct QSM from the raw imaging data.

### 2.3 QSM Generation

**FIGURE 1:**
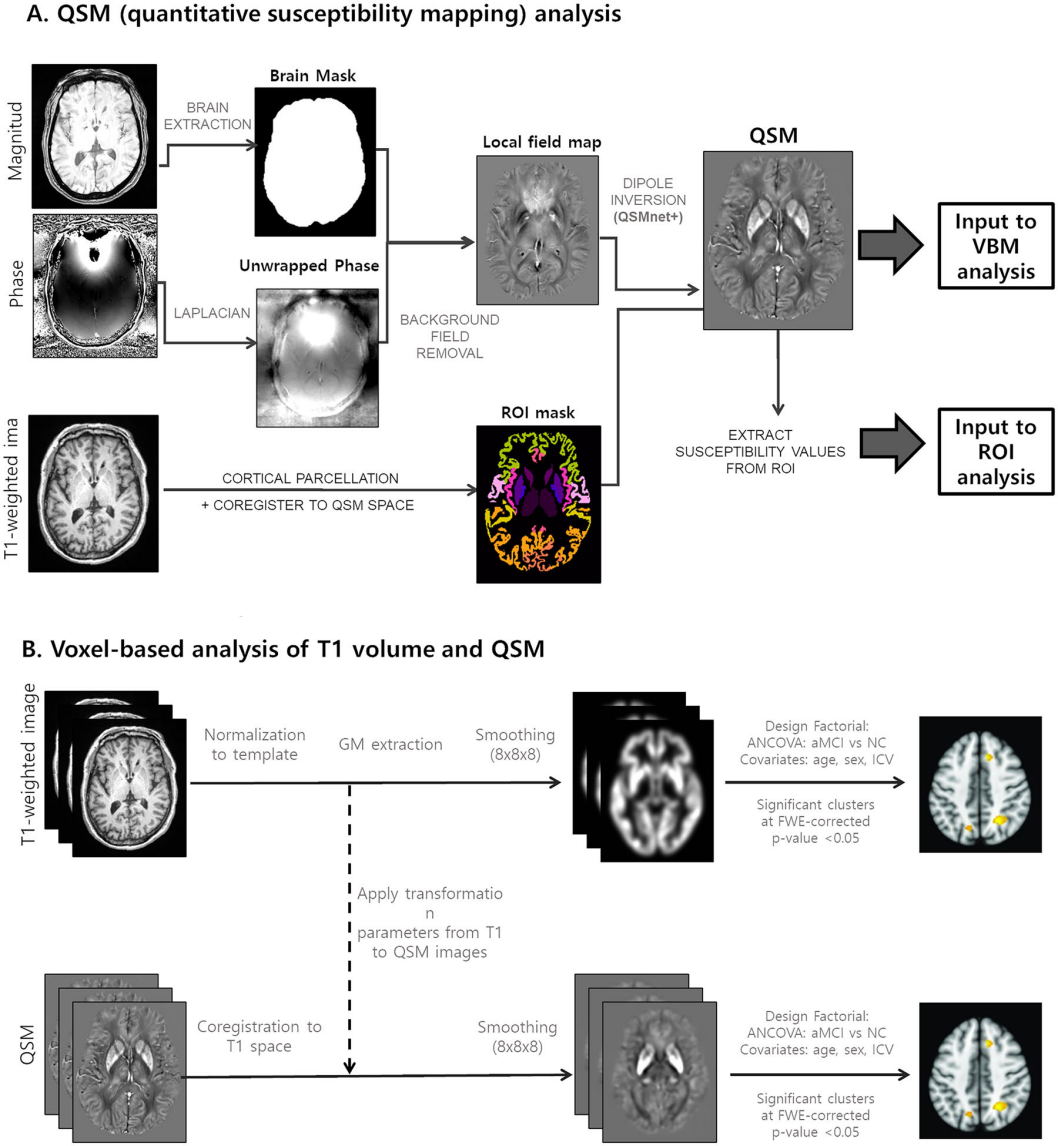
Overview of (A) QSM processing and (B) voxel‐based analysis of T1 volume and QSM.

QSM generation was demonstrated in Figure 1A. From the second echo of the GRE magnitude image, a brain mask was extracted using the Brain Extraction Tool (BET FSL, Oxford, UK) (S. M. Smith 2002). Within the brain mask, the GRE phase image was unwrapped using Laplacian phase unwrapping (Li et al. 2011). The background field was removed using V‐SHARP (B. Wu et al. 2012). From the remaining local field map, the QSM image was then reconstructed using QSMnet+ (Jung et al. 2020; Yoon et al. 2018), which is a neural network‐based dipole inversion method that has been validated to have good generalizability to a wide range of susceptibility values. To generate a normalized QSM map referenced to the CSF values, we divided the QSM map by the average lateral ventricular CSF values (Straub et al. 2017). Next, we performed voxel‐wise multiplication of the QSM and GM probability maps for each participant. This was performed so that the QSM value could be considered in relation to the GM density of that voxel, as QSM signals are also affected by susceptibility originating from the underlying white matter (WM) myelin. The GM‐weighted QSMs were used for voxel‐based and region‐of‐interest (ROI) analyses.

### 2.4 Voxel‐Based Analysis

For the VBM analysis, we used Statistical Parametric Mapping (SPM12) in MATLAB (MATLAB 2020a, The MathWorks, Inc). As shown in Figure 1B, T1‐weighted images were first spatially normalized to the SPM template and segmented into gray matter (GM) and WM tissue probability maps. Next, using the Diffeomorphic Anatomical Registration Through Exponentiated Lie Algebra toolbox (Ashburner and Friston 2000), we created a custom template based on GM and WM probability maps of the entire study population. This template was normalized to the standard Montreal Neurological Institute (MNI) template. The GM probability maps were then normalized to the MNI space with smoothing with 8 mm FWHM Gaussian filter. The Jacobian determinant maps are used to correct for any changes in volume that may have occurred during the registration process. For group comparisons, we employed an analysis of covariance (ANCOVA), factoring in age, sex, and intracranial volume as covariates. Normalized GM maps of the NC and aMCI groups were entered into the analysis. Statistical significance for ANCOVA was set at a *p*‐value < 0.05, while correcting for family wise errors (FWE). The statistical results were visualized using xjView 8.0 (Human Neuroimaging Lab). In the xjView software, information on which region the statistically significant clusters are concentrated in (where the peak value occurs) was derived as AAL3 v1 atlas labels (Rolls et al. 2020). For the QSM‐VBM analysis, steps similar to those described above were followed. The QSM was first co‐registered to the T1‐weighted image space using the affine matrix by co‐registering the second echo magnitude image to the T1‐weighted image. Next, the co‐registered QSM was spatially normalized to the MNI template by applying the transformation parameters obtained by spatially normalizing the T1‐weighted images. The QSM was then masked for the GM using a GM mask held at 0.5.

For the voxel‐based analysis, we analyzed all images from 109 participants instead of 81 participants to increase the statistical power to detect differences between the two groups. We used the voxel‐based QSM analysis from 109 participants to guide further exploration of notable regions using ROI‐based QSM analysis. Then in the remaining ROI‐based analyses, we included only 81 participants.

### 2.5 ROI‐Based Analysis

We conducted ROI analysis based on the AALv3 anatomical regions that corresponded to significant clusters in the voxel‐based analysis. T1‐weighted images were parcellated into anatomical regions using the Desikan–Killiany atlas on FreeSurfer v.6.0 (https://surfer.nmr.mgh.harvard.edu/). We used regions from the atlas that correspond to those identified in the AAL3 atlas from the voxel‐based analysis results: hippocampus, posterior cingulate, inferior parietal cortices, precuneus, cuneus, pericalcarine, and lateral occipital cortex. Using these ROIs, we extracted the regional volume and susceptibility measures.

### 2.6 Statistical Analysis

Statistical analyses were conducted using two software programs: MedCalc (version 15.2.2; Mariakerke, Belgium) and SPSS (IBM SPSS Statistics for Windows, version 21.0; IBM Corp., Armonk, NY, USA). After assessing the normality of the variables, clinical characteristics were compared using the independent samples *t*‐test for continuous variables and the chi‐squared test for categorical variables. Comparisons were made for GM atrophy and iron accumulation. For group comparisons, we also employed an analysis of covariance (ANCOVA), factoring in age, sex, and intracranial volume as covariates. Multiple comparisons were corrected for family wise errors (FWE). The association between QSM values and memory function scores was examined using Pearson's correlation. Following this, chosen ROI susceptibility values underwent stepwise linear regression to predict verbal (SVLT) or visual (RCFT) memory *z*‐scores, with age, sex, and education as controlled variables. A *p‐*value less than 0.05 was considered statistically significant.

## 3 Results

### 3.1 Basic Patient Characteristics

Forty‐eight aMCI patients and 33 age‐matched NC were included in the final analysis. The demographic characteristics are summarized in **Table** **1**. The mean age was 70.9 ± 5.8 years for NC and 71.3 ± 5.8 years for aMCI patients (*p* = 0.779). Patients with aMCI had lower MMSE scores (*p* < 0.001), higher depression scores (*p* = 0.034), and a higher proportion of APOE4 carriers (*p* = 0.001) than NCs. When comparing global and specific memory function, patients with aMCI showed significantly lower scores for both verbal memory (SVLT delay 1.52 ± 1.47 vs. 6.12± 2.50, *p* < 0.001) and visual memory test (RCFT delay, 6.29 ± 5.62 vs. 14.06 ± 5.60, *p* < 0.001) than NCs (Table 1).

**TABLE 1:** Clinicodemographic characteristics of the study population.

	NC (*n* = 33)	aMCI (*n* = 48)	*p* value
Age (years)	70.9 ± 5.8	71.3 ± 5.8	0.779
Female	23 (69.7%)	35 (72.9%)	0.752
Education (years)	11.3 ± 5.2	9.5 ± 5.2	0.129
Apoe4 positive	3 (9.1%)	21 (43.8%)	0.001
MMSE	27.9 ± 1.7	24.2 ± 3.7	< 0.001
SGDepS	3.0 ± 3.3	5.1 ± 4.0	0.034
History of depression	7 (15.2%)	6 (12.5%)	0.732

NC = normal control; aMCI = amnestic mild cognitive impairment; MMSE = Mini‐Mental State Examination; SGDepS = Short Geriatric Depression Scale.

*Note*: Data are presented as *n* (%) or as mean ± standard deviation.

### 3.2 Voxel‐Based Analysis

**FIGURE 2:**
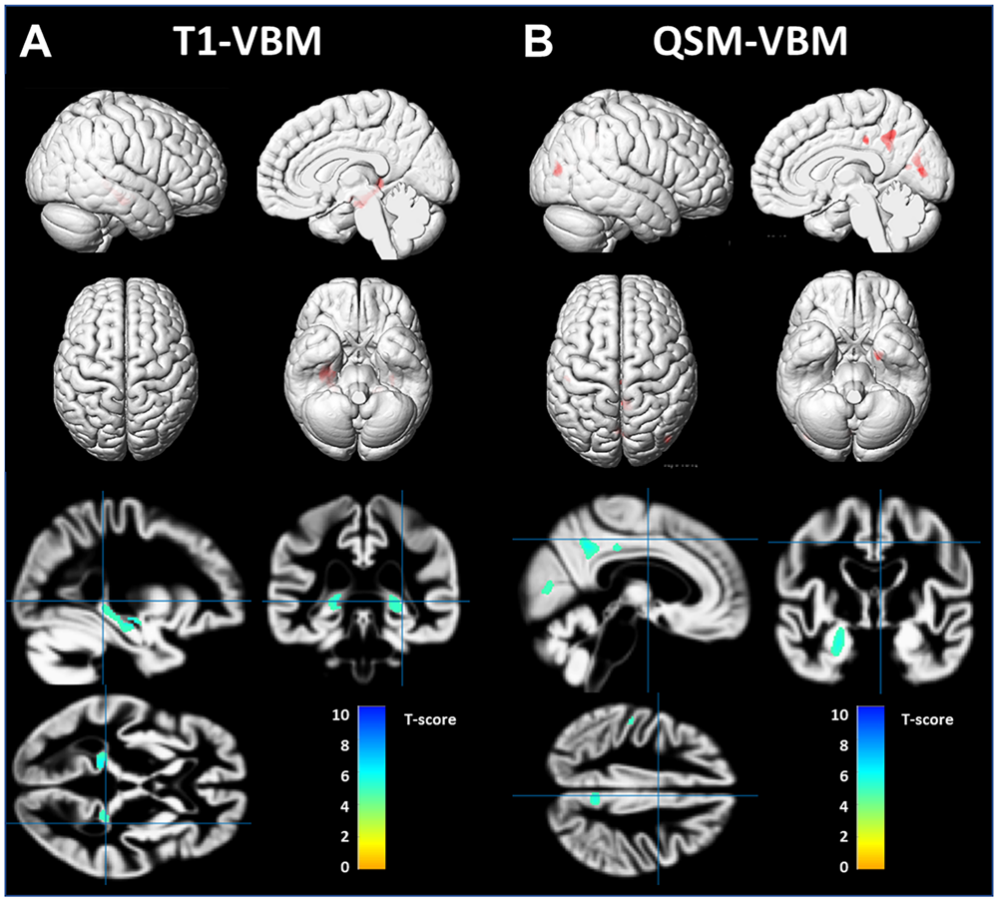
Patterns of GM atrophy (A) and iron accumulation (B) in patients with aMCI versus NC group. The results are shown on a 3D surface render (top) and overlaid on representative axial, coronal, and sagittal slices (bottom). Statistical significance of *p* < 0.05, corrected for multiple comparisons using few, are used. (A) T1‐VBM results: significant atrophy in the hippocampus in aMCI compared to NC group. (B) QSM‐VBM results: increased iron accumulation (noted as higher positive QSM values) in the amygdala/hippocampus, precuneus, posterior cingulate cortex, lateral occipital cortex, and pericalcarine cortex. Abbreviations: aMCI, amnestic mild cognitive impairment; CN, cognitively normal; GM, gray matter; QSM‐VBM, quantitative susceptibility mapping‐voxel‐based morphometry.

In 109 participants, voxel‐based analysis of GM volume showed significant bilateral hippocampal atrophy in the aMCI group compared to the NC group (FWE‐corrected *p*‐value < 0.05) (**Figure** **2A**).

In contrast, the voxel‐wise analysis of the QSM values showed significant differences to a wider extent in several regions. Compared to the NC group, the aMCI group showed higher QSM values in the right precuneus, left amygdala/hippocampus, bilateral calcarine cortex, right middle occipital cortex, right middle cingulate cortex, and left inferior parietal cortex (FWE‐corrected *p* < 0.05) (**Figure** **2B**). **Table** **2** shows the brain areas and MNI coordinates of significant clusters with a cluster size of at least 100 contiguous voxels.In 81 participants (age‐matched NC and aMCI) voxel‐based analysis found no statistically significant differences.

**TABLE 2:** VBM results using T1 volumetry and QSM.

	Brain region	Cluster size	MNI Coordinates			Peak *T* value
			*x*	*y*	*z*	
T1	Hippocampus_R	255	29	−12	−14	5.69
	Hippocampus_L	591	−16	−38	3	6.21
	Hippocampus_L	215	−33	−23	−9	5.61
						
QSM	Precuneus_R	985	3	−48	38	6
	Amygdala/hippocampus_L	838	−23	−5	−24	5.64
	Calcarine_L	505	0	−75	11	5.21
	Occipital_Mid_R	441	44	−80	12	5.43
	Calcarine_L	411	2	−76	10	5.28
	Cingulate_Mid_R	227	5	−27	38	5.2
	Parietal_Inf_L	139	−50	−26	42	5.37
	Calcarine_R	101	12	−65	13	5.02

### 3.3 Correlation Between Regional QSM Values and Verbal/Visual Memory Function

**FIGURE 3:**
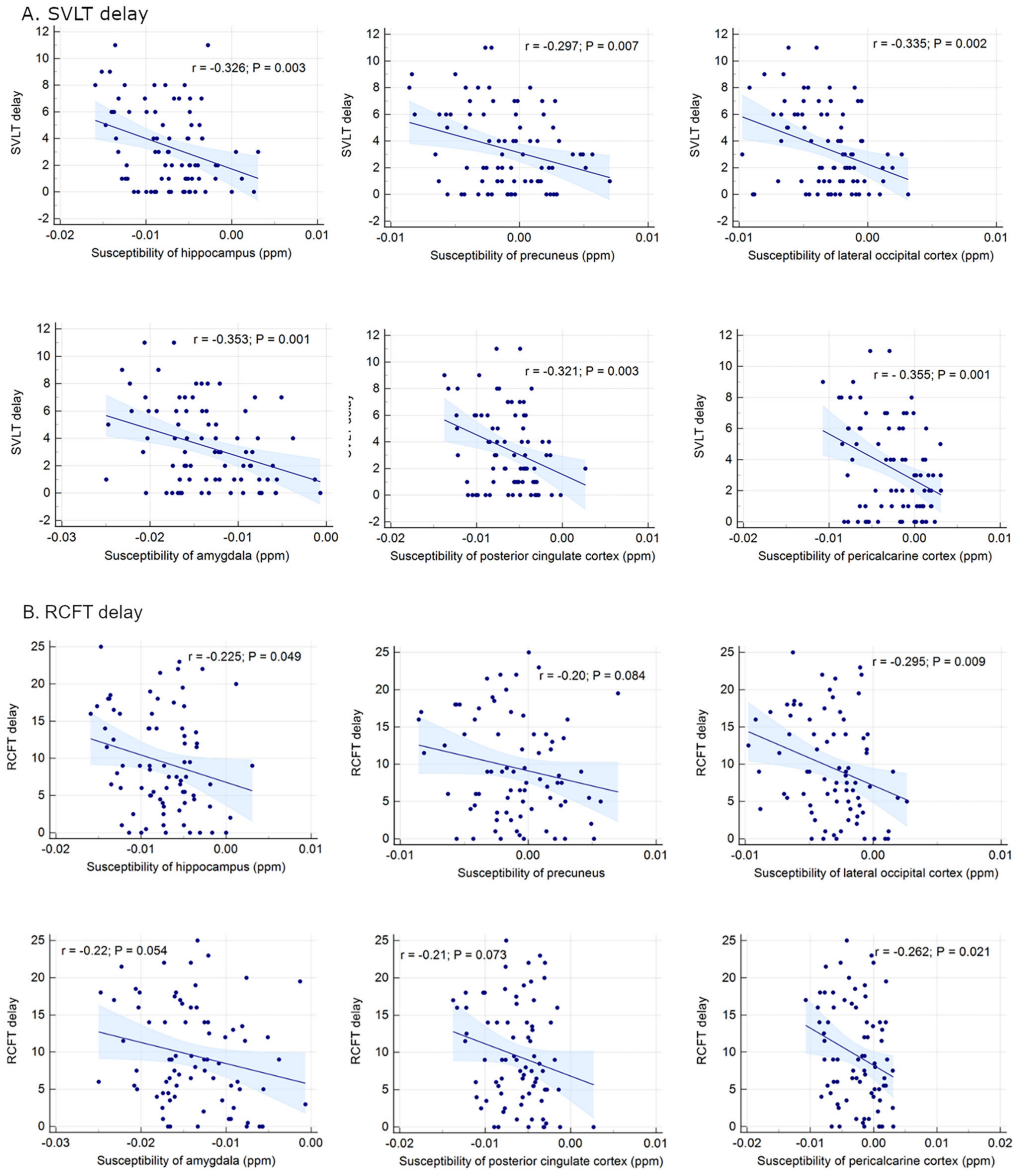
Correlation between the specific memory scores and the regional susceptibilities.

In the ROI‐based analysis (*n* = 81), the QSM values of the hippocampus (*r* = –0.326, *p* = 0.003), amygdala (*r* = –0.353, *p* = 0.001), precuneus (*r* = –0.297, *p* = 0.007), posterior cingulate cortex (*r* = –0.321, *p* = 0.003), lateral occipital cortex (*r* = –0.335, *p* = 0.002), and pericalcarine cortex (*r* = –0.355, *p* = 0.001) were negatively correlated with verbal memory score (SVLT). In contrast, the lateral occipital cortex (*r* = –0.295, *p* = 0.009), pericalcarine cortex (*r* = –0.262, *p* = 0.021), and hippocampus (*r* = –0.226, *p* = 0.049) were negatively correlated with the visual memory score (RCFT). The correlations were also demonstrated in Figure 3.

In aMCI group, the QSM value of the selected regions were not correlated with verbal/visual memory function. On the other than, in a NC group, the QSM values of the hippocampus (*r* = –0.418, *p* = 0.015), lateral occipital cortex (*r* = –0.484, *p* = 0.004), posterior cingulate cortex (*r* = –0.392, *p* = 0.024), and pericalcarine cortex (*r* = –0.444, *p* = 0.010) were negatively correlated with verbal memory score. In contrast, the QSM values of the selected regions were not correlated with visual memory function in NC group.

### 3.4 QSM Values as a Predictor of Specific Memory Dysfunction

In the study population (*n* = 81), stepwise multiple linear regression analysis showed that hippocampal volume (*B* = 903.95, SE = 442.11, *p* = 0.043), lateral occipital cortex susceptibility (*B* = –152.52, SE = 83.38, *p* = 0.070), and education (*B* = 0.320, SE = 0.054, *p* < 0.001) were predictors of MMSE scores (adjusted R2 = 0.313, *p* < 0.001).

Regarding verbal memory, stepwise multiple linear regression analysis revealed that a higher posterior cingulate cortex QSM value (*B* = –189.63, SE = 89.11, *p* = 0.037), lower hippocampal volume (*B* = 2015.91, SE = 469.61, *p* < 0.001), and lower education (*B* = 0.15, SE = 0.05, *p* = 0.007) were significant predictors of decreased verbal memory function (adjusted R2 = 0.306, *p* = 0.007), whereas QSM values of amygdala (*p* = 0.962), precuneus (*p* = 0.358), lateral occipital cortex (*p* = 0.425), and pericalcarine cortex (*p* = 0.815) were not.

For visual memory, only the higher susceptibility value of lateral occipital cortex (*B* = –659. 96, SE = 253.03, *p* = 0.011), lower education level (*B* = 0.046, SE = 0.13, *p* = 0.001), and male sex (*B* = –3.95, SE = 1.49, *p* = 0.010) were significant predictors of visual memory decline.

### 3.5 QSM Values as a Diagnostic Indicator of aMCI

In the study population (*n* = 81), logistic regression analysis revealed that amygdala susceptibility (*B* = 176.98, SE = 54.22, *p* = 0.001) was an independent diagnostic indicator of aMCI when controlling age, sex, education, hippocampal volume and the susceptibilities of other relevant regions (*p* = 0.0003). The receiver operating characteristic (ROC) curve of amygdala susceptibility for aMCI diagnosis was 0.727 (95% CI = 0.617–0.820), comparable to the ROC curve of hippocampal volume, which was 0.658 (95% CI = 0.544–0.760). A pair‐wise comparison of the ROC curves showed no significant difference (*p* = 0.285).

## 4 Discussion

Our study found that the QSM values were more widely affected than the volumetric measures in patients with aMCI. In our study population, smaller hippocampal volume, higher posterior cingulate cortical susceptibility, and lower education were predictors of verbal memory scores, whereas higher lateral occipital cortical susceptibility, lower education, and male sex were predictors of visual memory scores.

Although there has been no research on whether iron accumulation progresses in a sequent topographical pattern like amyloid and tau pathology, a large difference of involved regions, that is, various significant regions from the QSM‐VBM results and the single region from GM volume VBM of aMCI group results implies that the iron accumulation precedes brain atrophy. In a previous study with a smaller sample size (H. G. Kim et al. 2017), QSM values in the aMCI group were significantly higher in the precuneus, allocortex and cingulate cortex compared to the CN group, whereas gray matter volume differed significantly only in the hippocampus between the two groups. These findings are consistent with our results. Our findings are also in line with those of a previous study suggesting that iron accumulation are better associated with cognitive scores than volumetric measures in cognitively normal individuals (Chen et al. 2021). Chen et al. reported that elevated brain iron levels by QSM were associated with lower cognitive performance independent of beta‐amyloid accumulation by PET (Chen et al. 2021). Beta‐amyloid accumulation is regarded as the most upstream event in neurodegeneration, such as neuronal and synaptic loss. Our findings imply that the accumulation of iron measured by QSM may play a role as an early imaging correlate compared to brain volume measure in AD and cognitive impairment (H.‐G. Kim et al. 2017). It's also possible that iron measurements are simply more sensitive markers than brain volume measurements.

Further iron accumulation measured by QSM may not be just another facet of nonspecific neurodegeneration. Accumulating evidence suggests the brain iron accumulation is linked to ferroptosis, which is suggested as one of the major causes of AD‐related neurodegeneration. Iron may interact with beta‐amyloids, triggering essential pathological processes that result in AD. In situations where amyloid levels are abnormal, amyloid can attach to ferric iron (Fe^3+^) and transform it into its redox‐active variant, ferrous iron (Fe^2+^). It interacts with hydrogen peroxide to create hydroxyl radicals, causing oxidative damage (Chiang 2021). Excessive brain iron accumulation is also associated with ferroptosis, a newly recognized nonapoptotic iron‐dependent cell death mechanism (Ren et al. 2020). In detail, excessive iron induces lipid peroxidation, which lead to an oxidative cell death. In preclinical studies, ferroptosis has been considered an important sign of AD and cognitive impairment (Chen et al. 2015; Hambright et al. 2017).

We found elevated iron levels in the amygdala, hippocampus, right precuneus, right middle cingulate cortex, left inferior parietal cortex, and bilateral occipital cortex in the aMCI group compared with those in the NC group. Consistent with our findings, accumulation of iron in the hippocampus has been observed in the early stages of AD (Ding et al. 2009; Leskovjan et al. 2011; Raven et al. 2013). Patients with AD have been found to exhibit increased levels of ferritin iron accumulation in the hippocampus, which is associated with hippocampal damage, compared with healthy controls. Moreover, the degree of iron deposition is correlated with the disease duration (Ding et al. 2009; Raven et al. 2013). Consistent with these findings, our results showed that the MNI coordinates of GM VBM and QSM‐VBM overlapped in over 100 clusters in the hippocampus. Iron accumulation areas beside the hippocampus are consistent with known amyloid pathology‐prone areas. The precuneus, cingulate cortex, and parietal cortex are well‐known to be involved in early amyloid accumulation (Mattsson et al. 2015; Palmqvist et al. 2017). Excess iron exacerbates the accumulation of toxic amyloid (Mattsson et al. 2015; Palmqvist et al. 2017) and tau (Sayre et al. 2000). In a recent study with subcortical vascular MCI patients, the smaller volume and the higher susceptibility of cingulate region was observed than cognitively normal individuals (Liu et al. 2025). The additional elevated brain iron in the occipital cortices in the aMCI versus NC group in our study is in line with a previous study that found more pronounced iron accumulation in the temporal and occipital lobes, but not in the other lobes (Damulina et al. 2020).

The inverse relationship between the verbal memory score and elevated iron levels in the hippocampus/amygdala is consistent with previous reports. A relationship between increased hippocampal iron levels and reduced memory performance has been consistently reported (Spence et al. 2020). However, the relationship between cortical iron and verbal/visual memory has not been elucidated using in vivo imaging studies. The significant inverse relationship between verbal memory function and iron accumulation in the precuneus, posterior cingulate cortex, lateral occipital cortex, and pericalcarine cortex corresponds to the established brain regions related to verbal memory function (Emch et al. 2019). Similarly, poor visual memory function is associated with elevated iron accumulation in the lateral occipital and pericalcarine cortices, which are associated with visual working memory (Ishai et al. 1999; Pessoa et al. 2002).

We believe that the detrimental effect of increased iron levels on verbal memory may be related to two different mechanisms. The relationship between hippocampal iron and verbal memory suggests that iron accumulation might be related to tau pathology or ferroptosis itself, rather than amyloid pathology (Vogels et al. 2020). By contrast, elevated iron levels in the cingulate/precuneus may reflect amyloid‐dependent pathology (Grothe et al. 2017). On the other hand, underlying mechanism of the relationship between occipital cortex and visual memory remains unclear. Considering that occipital lobe is the primary brain region for visual processing and plays a role in storing visual information related to episodic memory (Geissmann et al. 2023), this relationship may reflect iron accumulation in the affected area due to neurodegenerative process.

Although these negative correlations between QSM values and memory‐related neural correlates do not cover every known memory‐functioning region, such as the frontoparietal areas, the spatial correspondence of verbal and visual memory‐related brain regions and iron accumulation suggests that QSM values can be used as a new objective marker for verbal/visual memory dysfunction. Memory dysfunctions are difficult to assess in the early stages of the disease and require complex and comprehensive neuropsychiatric tests. Therefore, this new radiological diagnosis based on the QSM value raises the expectation that it would simplify and advance the diagnostic step of verbal and memory dysfunction. Furthermore, our multiple regression analysis independently identified iron accumulation in the posterior cingulate cortex as a predictor of verbal memory decline. The presence of higher iron accumulation was particularly prominent in the posterior cingulate cortex and lateral occipital cortex, regions that show early amyloid‐beta plaque accumulation and subsequent neurofibrillary tangle deposition in preclinical and clinical AD studies (Hwang et al. 2021; Insel et al. 2020; Palmqvist et al. 2017). Both postmortem and in vivo studies have demonstrated a spatial correlation between iron accumulation and amyloid or tau aggregation (Everett et al. 2014; Sayre et al. 2000), suggesting that iron accumulation may be involved in the pathogenesis of early AD by accumulating in senile plaques and neurofibrillary tangles, where oxidative stress induced by a redox imbalance can cause neuronal degeneration (Everett et al. 2014; M. A. Smith et al. 2010).

QSM values in the posterior cingulate cortex may serve as predictors of verbal memory dysfunction, as previous studies have found that amyloid accumulation in this region is associated with increased susceptibility in individuals with aMCI (H. G. Kim et al. 2017). Furthermore, high QSM values in the posterior cingulate cortex have been shown to correlate with tau aggregation in the temporal lobe, further supporting the use of QSM values in this region as predictors of cognitive decline (Spotorno et al. 2020). Overall, the higher positive QSM values observed in the posterior cingulate cortex and lateral occipital cortex may be useful predictors of decreased verbal and visual memory functions, respectively, and highlight the potential of iron accumulation as a biomarker for early AD detection. Iron accumulation represented by QSM is a multifaceted process influenced by various biological factors. In AD and aMCI, several mechanisms have been proposed to explain this phenomenon. Normal brain function relies on a delicate balance of iron levels, and the balance is regulated by proteins such as transferrin, ferritin, and ferroportin. In aMCI, this regulatory system may become disrupted and lead to excessive accumulation of iron (M. A. Smith et al. 1997). In another study, blood–brain barrier (BBB) breakdown has been observed, which could allow excess iron to enter the brain from the systemic circulation. This process might be facilitated by inflammatory mediators and oxidative stress which are common features in AD pathology (Bell and Zlokovic 2009). In addition, iron has been shown to interact with amyloid‐beta and tau. Amyloid plaques and neurofibrillary tangles can bind iron, and lead to localized iron accumulation and further oxidative stress and neuronal damage (Honda et al. 2004). Finally, our result indicate that the susceptibility of amygdala can differentiate aMCI from CN, with an ROC of 0.727 (95% CI = 0.617–0.820), which is comparable to the ROC of hippocampal volume (0.658, 95% CI = 0.544–0.760). These findings suggest that QSM may serve as an adjunct diagnostic indicator for aMCI, particularly in cases where 3D T1 volumetric images for hippocampal measurement are unavailable. However, further validation in large cohorts is needed to establish QSM as an independent diagnostic tool.

Our study has a few limitations. First, the cross‐sectional nature of this study has limitations in exploring the accurate temporal relationship between volume measurements and QSM values during disease progression. Second, we did not incorporate amyloid or tau PET data in this retrospective study. Incorporating amyloid and/or tau PET would be helpful for evaluating the interactions between brain volume atrophy, iron accumulation, and memory decline in a more precise manner. In addition, we did not analyze APOE4 gene effect in this study because of a relatively small sample size. Future studies with larger sample sizes and longitudinal design are warranted to examine this association more thoroughly.

In conclusion, our findings suggest that iron accumulates in areas where atrophy has not yet occurred, and that iron may be an earlier marker of neurodegeneration or a more sensitive marker of neurodegeneration. Further studies are needed to investigate the longitudinal relationship between brain volume and iron accumulation during cognitive decline.

## Author Contributions

**Subin Lee**: Writing—review and editing, formal analysis, conceptualization, data curation, investigation, methodology, software, validation, visualization, writing—original draft. **Suhyeon Lee**: Writing—review and editing, formal analysis. **Ina Park**: Formal analysis, investigation, writing—review and editing. **Yeonsil Moon**: Writing—review and editing, validation, investigation, data curation. **Younghee Yim**: Formal analysis, investigation, validation, writing—review and editing. **Jongho Lee**: Resources, methodology, writing—review and editing. **June Sic Kim**: Writing—review and editing, validation. **Won‐Jin Moon**: Conceptualization, investigation, funding acquisition, writing—original draft, writing—review and editing, project administration, supervision, data curation, formal analysis, validation.

## Conflicts of Interest

The authors declare no conflicts of interest related to the content of this article.

## Ethics Statement

This retrospective study, identified as IRB No. 2020‐09‐030, was approved by the Institutional Review Board of Konkuk University Medical Center. The research was conducted in full compliance with the principles of the Declaration of Helsinki. Due to the retrospective nature of the study, the requirement for informed consent was waived by the Institutional Review Board. All patient data were anonymized and handled with strict confidentiality.

### Peer Review

The peer review history for this article is available at https://publons.com/publon/10.1002/brb3.70521

## Data Availability

Research data are not shared.
